# Two *Salix* Genotypes Differ in Productivity and Nitrogen Economy When Grown in Monoculture and Mixture

**DOI:** 10.3389/fpls.2017.00231

**Published:** 2017-02-21

**Authors:** Stefanie Hoeber, Petra Fransson, Inés Prieto-Ruiz, Stefano Manzoni, Martin Weih

**Affiliations:** ^1^Department of Crop Production Ecology, Swedish University of Agricultural SciencesUppsala, Sweden; ^2^Department of Forest Mycology and Plant Pathology, Swedish University of Agricultural SciencesUppsala, Sweden; ^3^Department of Plant Protection, Institute of Agricultural Sciences – Spanish National Research CouncilMadrid, Spain; ^4^Department of Physical Geography, Stockholm UniversityStockholm, Sweden; ^5^Bolin Centre for Climate Research, Stockholm UniversityStockholm, Sweden

**Keywords:** plant competition, biomass allocation, biomass production, willow, nitrogen use efficiency, community level, *Salix*

## Abstract

Individual plant species or genotypes often differ in their demand for nutrients; to compete in a community they must be able to acquire more nutrients (i.e., uptake efficiency) and/or use them more efficiently for biomass production than their competitors. These two mechanisms are often complementary, as there are inherent trade-offs between them. In a mixed-stand, species with contrasting nutrient use patterns interact and may use their resources to increase productivity in different ways. Under contrasting nutrient availabilities, the competitive advantages conferred by either strategy may also shift, so that the interaction between resource use strategy and resource availability ultimately determines the performance of individual genotypes in mixtures. The aim was to investigate growth and nitrogen (N) use efficiency of two willow (*Salix*) genotypes grown in monoculture and mixture in a fertilizer contrast. We explored the hypotheses that (1) the biomass production of at least one of the involved genotypes should be greater when grown in mixture as compared to the corresponding monoculture when nutrients are the most growth-limiting factor; and (2) the N economy of individual genotypes differs when grown in mixture compared to the corresponding monoculture. The genotypes ‘Tora’ (*Salix schwerinii* ×*S. viminalis*) and ‘Loden’ (*S. dasyclados*), with contrasting phenology and functional traits, were grown from cuttings in a growth container experiment under two nutrient fertilization treatments (high and low) in mono- and mixed-culture for 17 weeks. Under low nutrient level, ‘Tora’ showed a higher biomass production (aboveground biomass, leaf area productivity) and N uptake efficiency in mixture than in monoculture, whereas ‘Loden’ showed the opposite pattern. In addition, ‘Loden’ showed higher leaf N productivity but lower N uptake efficiency than ‘Tora.’ The results demonstrated that the specific functional trait combinations of individual genotypes affect their response to mixture as compared to monoculture. Plants grown in mixture as opposed to monoculture may thus increase biomass and vary in their response of N use efficiency traits. However, young plants were investigated here, and as we cannot predict mixture response in mature stands, our results need to be validated at field scale.

## Introduction

Nutrients are among the most limiting factors for many plants grown in managed and natural ecosystems, therefore nutrient availability is often a main driver for plant adaptation and acclimation patterns. Thus plant nutrient uptake rates depend partly on the soil nutrient availability and partly on the capacity of the plant to acquire available nutrients and allocate them to different tissues and functions. As a consequence, plants often adopt different strategies to acquire and allocate nutrients depending on resource availability, ranging from efficient utilization of limited nutrients for biomass production to intensive nutrient uptake (Grime, 1977). The so-called ‘competitors’ are able to capture and deplete limited resources rapidly, by having a flexible morphology and/or maintaining high growth rate under moderate stress. These competitors are predicted to have traits that are advantageous in fertile environments leading to rapid growth and high resource capture, such as rapid turnover of leaves, short leaf lifespan and high leaf nutrient concentration; but also high nutrient losses and low nutrient retention (Aerts, 1999). In contrast, ‘stress tolerant’ species characterized by slow turnover of leaves, long tissue lifespan and low leaf nutrient concentration (i.e., properties that increase high nutrient retention) may be more successful than competitors in nutrient-limited environments.

These resource acquisition strategies can be mapped into sets of functional traits that link plant growth pattern to specific processes. In addition, resource acquisition pattern impacts fitness indirectly via its effects on growth, reproduction and survival (Violle et al., 2007; Craine, 2009). Depending on nutrient availability, the values of the traits controlling resource acquisition and growth may vary (Aerts, 1999). For example under nutrient limitation, many plants tend to allocate available resources predominantly to roots to reach mineral nutrients (Bloom et al., 1985; Aerts, 1999). In contrast, at high nutrient availability, roots of inherently fast-growing plants have a capacity to rapidly increase their nutrient uptake capacity (Jackson and Caldwell, 1996), and biomass allocation shifts to shoots and leaves is prioritized (Lambers and Poorter, 1992). Having a great capacity for rapid growth and nutrient acquisition when nutrient resources are in sufficient supply is often correlated with a low efficiency to use the acquired nutrients for biomass production and vice versa (Hobbie, 1992; Reich, 2014). Patterns of variation in these two mechanisms – nutrient uptake efficiency and the efficiency of nutrient conversion to biomass production (sometimes called nutrient utilization efficiency or nutrient productivity) – describe the plant nutrient economy. Calculation of these traits is most meaningful in situations in which nutrients are among the most limiting factors for plant production, where they can be used as indicators for changes in nutrient economy when comparing plants grown in monocultures with mixed stands, and across nutrient availability gradients.

Plants grown in mixed stands, involving species or genotypes with potentially contrasting nutrient economy (e.g., different nutrient uptake rate, tissue nutrient concentration, nutrient translocation efficiency), may show growth patterns that differ from those in monocultures, because they interact with plants characterized by more diverse strategies. In general, two mechanisms may increase productivity in mixed stands compared to monocultures: the sampling (or selection) effect by which community components with specific functional traits are selected, and niche differentiation (complementarity effect) by which resource exploration patterns of community components vary in space and time (Tilman et al., 1997; Loreau, 1998, 2000). The sampling effect is based on the idea that the community components (i.e., species or genotypes) with the highest resource (e.g., nutrient) uptake efficiency outcompete the others in the long term, because they lower the nutrient level in the soil of a mixed stand most (Tilman et al., 1997; Loreau, 2000). The sampling effect therefore acts through changed population dynamics over time and is based on functional trait differences between species or genotypes related to resource acquisition and growth. Functional trait differences between species or genotypes are important also for niche differentiation, but are here linked to resource exploitation in different spatial niches or time periods (i.e., temporal niches), including growth processes over short and long time scales. The niche differentiation effect is thus suggested to improve the whole community productivity (Hooper and Vitousek, 1997; Tilman et al., 1997; Loreau and Hector, 2001). When the lifetime of a plant community is limited (e.g., to one growing season), and the community composition is defined *a priori* (e.g., by the experimental design), the interaction effects resulting from niche differentiation can be studied by assessing how plant traits related to growth and nutrient economy vary in the community components across different nutrient availabilities and community compositions. A promising approach is the trait-based (e.g., growth and nutrient economy traits) comparison of species or genotypes when grown in monoculture and mixed stands. Community dynamics have hitherto mostly been studied using different species as community components; and trait-based approaches including functionally different genotypes are rare.

Plant community dynamics related to the resource use– productivity–diversity interaction have frequently been studied across taxonomically different tropical and temperate trees and grasslands (Erskine et al., 2006; Morin et al., 2011). While most work has focused on slow vs. fast growing species, less attention has been devoted to boreal tree species or taxonomically closely related tree species. Here, we focus on the productivity and nutrient use in two different willow (*Salix* spp.) genotypes (‘Loden’ and ‘Tora’) with contrasting phenological characteristics and nitrogen (N) use (Weih and Nordh, 2002). Specifically, ‘Tora’ is known to have a higher productivity, root growth rate and leaf N concentration, but lower leaf area ratio and leaf area productivity (LAP), compared to ‘Loden.’ In general, *Salix* species are fast-growing trees and efficient N users, generally achieving a high biomass at low N availability, making them suitable for biomass production in short-rotation coppice systems (Karp and Shield, 2008). Furthermore, due to their high genetic variation in phenological traits, *Salix* plants adapt to latitudinal changes and can be grown under Mediterranean to boreal climate with different nutrient resource availability (Weih, 2009; Bonosi et al., 2013). These features make willow a good model to test the hypothesis that mixtures are more productive than monocultures in resource-limited environments; and to investigate the relevant growth and N economy traits of the individual community components in relation to their effects on niche differentiation when grown in mixture. It is known that *Salix* plantations consisting of a mixture of many genotypes often produce more biomass than most of the corresponding *Salix* monocultures in the long term (Dawson and McCracken, 1995), but this effect is likely to be caused by the different susceptibility of community components to pathogens affecting the population dynamics over longer periods of time, and thus beyond the niche differentiation focus applied here. In our study, we expect that higher productivity can be achieved by two mechanisms: (1) the biomass production of at least one of the involved community components, i.e., genotypes, should be greater when cultured in mixture as compared to the corresponding monoculture when nutrients are the most growth-limiting factor; and (2) the N economy of the genotypes differs when cultured in mixture compared to the corresponding monocultures. These hypotheses are tested at the individual plant level and at the whole community level in a growth container experiment to minimize confounding factors like individual tree age and size, and spatial heterogeneities occurring in natural settings. Our setup thus allowed isolating the effect of functional trait diversity among genotypes in relation to niche differentiation when individual genotypes are grown in mixture; and calculating complete N budgets in different biomass compartments for each genotype in both mixtures and monocultures.

## Materials and Methods

### Experimental Setup

The experiment was carried out outdoors in a roofed net-enclosed yard at Ultuna campus, Uppsala, Central Sweden (59°49′ N 17°39′ E). Two different *Salix* genotypes; ‘Tora’ (*S. schwerinii* E. Wolf *× S. viminalis* L.*)* and ‘Loden’ (*S. dasyclados* Wimm.), were grown either in monoculture or in genotype mixture (50:50). Five-cm cuttings of the two genotypes were randomly selected for potting. Diameter and weight of each cutting were recorded. Before planting, cuttings were put into tap water for 2 days to soak. A total of six cuttings were planted in 16.9 L rectangular pots with four 6 mm holes (one per side) drilled at 20 mm height from the bottom, lined with cellulose fabric and filled with 22 kg washed quartz sand (Specialsand 0.17 mm, Råda sand AB, Lidköping, Sweden) on May 21, 2014. Cuttings were planted at an equal distance to the nearest neighbor, with 0.1 m distance to each other. In pots containing genotype mixtures, three cuttings per genotype were planted systematically in the following order; row one ‘Loden,’ ‘Tora,’ ‘Loden,’ and row two ‘Tora,’ ‘Loden,’ ‘Tora.’ After planting, all pots were watered until soil saturation, and then watered with tap water every 2–3 days for 1 month until the irrigation treatment was started in June 24.

The experiment had a full-factorial block design: two nutrient (F- and F+) and two irrigation (W- and W+) treatments for the ‘Loden’ and ‘Tora’ monocultures and ‘Loden’ – ‘Tora’ mixtures in four blocks, giving a total of 12 pots per block (in total 48 pots and 288 plants). An additional two pots planted with six cuttings of genotypes ‘Tora’ and ‘Loden’ in monoculture, respectively, were used for an initial plant harvest. Fertilizer treatments started after 1 month of growth, when also an initial harvest was conducted, as in Weih and Nordh (2002). To achieve total N inputs of 20 (F-) and 120 kg N ha^-1^ year^-1^ (F+), plants were supplied with 1.4 (F-) and 8.4 mg N week^-1^ (F+) added to the irrigation during the main growth period from mid-June until mid-September. The regular (i.e., weekly) addition of nutrients over the main growing season ensured nearly steady-state conditions for the plants, and no signs of nutrient deficiency were observed during the entire course of this experiment. Nutrient solutions were made from the full- nutrient solution ‘Blomstra’ (Cederroth, Upplands Väsby, Sweden) with NH_4_^+^ and NO_3_^-^ nitrogen in proportion 19:32 and N, P, K, and Mg in the proportion 50:10:45:3.

For the irrigation treatment, plants in the well-watered treatment (W+) received 600 ml fertilized water every third day as a regular watering scenario. Plants in the water-stressed treatment (W-) were exposed to the same total amount of water as in the W+ treatment, but received 1200 ml fertilized water every sixth day to simulate more intense rainfall events interspaced by longer dry periods (a ‘climate change’ rainfall scenario). We expected an interaction between the watering and fertilization treatments caused by increased nutrient leaching under the ‘climate change’ rainfall regime (W- treatment). All pots were rotated every second day during the whole growth period to minimize effects of position on plant performance. The mean air temperature and relative humidity were recorded by Ultuna meteorological station. Mean temperature between initial and final harvest was 16.9°C with a minimum temperature at 3.5°C and maximum temperature at 33°C. Mean relative humidity during that period was 72.1%, with maximum and minimum relative humidity at 98% and 22.9%, respectively. Mean maximum photosynthetically active radiation during the main growth period was 1251 μmol m^-2^ s^-1^, which is approximately 62% of the mean maximum measured solar radiation.

### Measurement of Productivity and Allocation of Plant Material

Two destructive plant harvests were conducted: an initial harvest in June 24 after 34 days of growth when plants sprouted and showed 1–2 leaves (one pot with six plants per genotype), and the final harvest in September after 120 days of growth before leaves entered the senescence stage (full treatment design). At harvest, plants were separated into leaves, shoots, cuttings, and roots. Leaves were counted and total plant leaf area was scanned and analyzed with WINDIAS 2.0 (Delta-T Devices, Cambridge, UK). Shoot height and diameter were recorded, and cuttings were washed in tap water. All aboveground plant parts (for each individual) were oven-dried at 70°C for 48 h and dry weight was recorded. Specific leaf area (SLA, m^2^ kg^-1^) was calculated for individual plants by dividing leaf area by leaf weight. Pots with root samples were initially stored in a freezer at –18°C until further processing. Before handling, the pots with the root samples were put into a fridge at 4°C for 24 h for defrosting and subsequently cleaned from sand and washed with tap water. In total, root systems of two replicate pots (six plants per pot, and two pots per treatment) were washed. From two out of six plants per pot in monocultures and mixtures, three root fragments with the size of 0.1 m were scanned to measure specific root area. Total N content was determined by dry combustion (Dumas principle, ISO 13878) by pooling samples as follows. All leaves for each plant and genotype were pooled in each pot (three plants per genotype in mixture, six plants in monoculture). Moreover, two replicates per genotype for shoots and roots were also pooled for each pot. Total N concentrations were finally calculated multiplying N content with the weight of each plant organ. In this way, total N content and concentration measurements are representative of each genotype growing in each pot.

### Diversity Effects

The relationship between aboveground biomass in monoculture and mixture can be analyzed by using an additive partitioning method (Loreau and Hector, 2001). Following this method, net diversity effects are evaluated from the differences between the observed aboveground biomass in the mixture and the expected aboveground biomass for the mixtures based on the individual performances in the respective monocultures. The net diversity effect was calculated as the sum of the complementarity and selection effect. A positive selection effect implies that species performing best in monocultures are dominant in mixture, whereas a negative selection effect indicates that genotypes performing less well in monoculture are advantaged in mixture. Positive complementarity effect occurs when the genotypes perform together better in mixture than observed in monoculture.

To calculate the diversity effect with a representative number of monoculture and mixture combinations, we selected random pairs of aboveground biomass values from our seven to eight replicated mixture and monoculture pots. In total, 300 combinations were obtained to span the spectrum of combinations among the replicated pots. The same procedure was adopted for both fertilization treatments. Based on these paired values, the diversity effects were partitioned between selection and complementarity following Loreau and Hector (2001). Finally, we averaged the diversity effects across replicates, for each combination from the random extraction procedure. The average values were then used for subsequent statistical analysis.

### Data Analysis

Plant growth and N economy of *Salix* plants were based on an initial harvest and a final harvest (before leaves entered the senescence stage). We calculated growth parameters at the genotype level and for each pot using classical growth analysis as described in **Table 1** (Fisher, 1921; Evans, 1972; Hunt, 1982). Plant N economy was calculated based on the methodology by Weih et al. (2011) and Weih (2014); using the N content of the cutting as perennial N (i.e., *N*_s_), and the N contents of the plants from the two destructive harvests for the calculation of the mean plant N content during major growth period (*N’*). As we had controlled N inputs and N fate in the plant biomass, it was also possible to evaluate the community level N retention efficiency (η) as the fraction of N retained in the system by the end of the growing period. Some parameter values were log_e_-transformed or square root transformed prior to statistical analysis to achieve normality. With the irrigation treatment, or the so called ‘climate change’ scenario with long dry periods, no water stress developed (see Supplementary Material). Hence, we focused statistical analyses on the fertilization and mix-mono culture treatments, but utilized the data from the irrigation treatments as additional replicates (resulting in *n* = 4 or 8 depending on measurement). All statistics were conducted with the software package R (Version 3.0.3). A linear mixed model was used to assess the effects of fertilization treatment, genotype and mix-mono culture, with pot used as a random factor [lme package: ‘*nlme’* by Pinheiro et al. (2014)]. In addition, a linear mixed model was also used to assess all two-way and three-way interactions between fertilization treatments, genotypes and mix-mono culture. To test the effect of fertilization, genotype and mix-mono culture on pot (community) level, an analysis of variance (ANOVA) was conducted with Tukey HSD test as a *post hoc* test [glht package: ‘multcomp’ by Hothorn et al. (2008)]. Analysis of Covariance (ANCOVA), with the natural logarithm of total plant biomass as a covariate, was used to analyze differences in root allocation. For each diversity effect (net diversity, complementarity, and selection effect), a two-tailed Student’s one-sample *t*-test was performed to assess if diversity effects were significantly different from zero. Since the number of combinations (300) is arbitrary and significance is related to the number of samples, the same procedure was repeated with 10 and 50 iterations, leading to the same significant effects.

**Table 1:** Trait abbreviations, definitions, and units.

Abbreviation	Unit	Trait	Definition/equation
AGB	g	Aboveground biomass	Sum of aboveground biomass
E_N,y_	g g^-1^	Yield specific N efficiency	m_shoot_/(plant N_initial_+plant N_harvest_)/2
LAP	g m^-2^ wk^-1^	Leaf area productivity	N_leaf_/A_leaf_ × LNP
LAR	m^2^ g^-1^	Leaf area ratio	A_leaf_/m_total_
LMR	g g^-1^	Leaf biomass fraction	m_leaf_^/^m_total_
LN/LA	mmol N m^-2^	Leaf N content	N_leaf_/A_leaf_
LNP	g [mol N]^-1^ wk^-1^	Leaf N productivity	(log_e_N_leaf,2_ – log_e_N_leaf,1_)/(N_leaf,2_ – N_leaf,1_) × (m_leaf,2_ – m_leaf,1_)/(*t*_2_–*t*_1_)
RGR	g g^-1^ wk^-1^	Relative growth rate	(log_e_ m_leaf,2_ – log_e_ m_leaf,1_)/(*t*_2_–*t*_1_)
RMF	g g^-1^	Root mass ratio or root mass fraction	m_root_/m_total_
Root:shoot	g g^-1^	Root to shoot ratio	m_root_/m_shoot_
SLA	m^2^kg^-1^	Specific leaf area	A_leaf_/m_leaf_
SRA	cm^2^ g^-1^	Specific root area	A_root_/m_root_
SRL	m g^-1^	Specific root length	L_root_/m_root_
U_N_	g g^-1^	N uptake efficiency	(plant N_initial_ + plant N_harvest_)/2/N_cutting_
η	g g^-1^	N retention efficiency	(plant N_harvest_ – plant N_initial_)/N_fertilizer_

*Dry weights of the biomass are indicated by ‘m,’ area by ‘A,’ and nitrogen content (mass or mol) by ‘N’; subscripts refer to plant organs.*

## Results

Plant biomass traits, the traits associated with productivity, and the traits related to N acquisition and allocation are presented first at the individual plant level (a single plant of a given genotype is used as a unit; Sections “Plant Biomass Allocation and Productivity” and “Nitrogen Economy”) and then at the community level (at which the whole pot is used as a unit; Sections “Aboveground Biomass, N Economy, and N Retention Efficiency at the Plant Community Level” and “Diversity Effects”).

### Plant Biomass Allocation and Productivity

**FIGURE 1:**
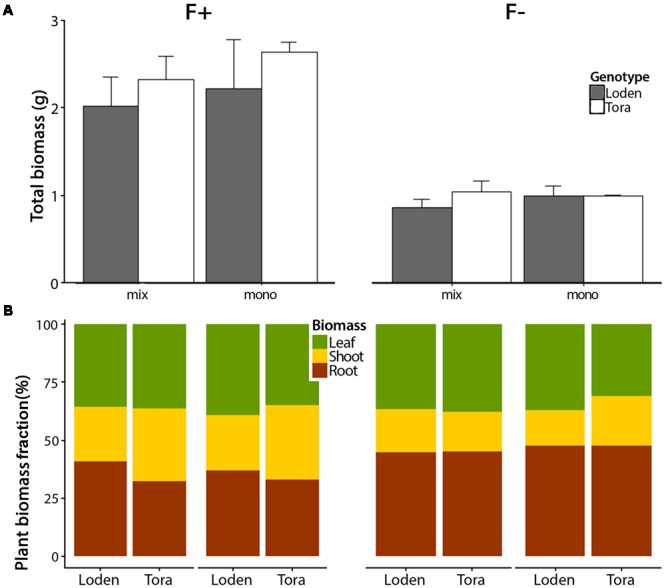
**Biomass allocation of leaves, shoots, and roots between two *Salix* genotypes grown in two fertilizer and mono-mix treatments. (A)** Mean total biomass (leaf, shoot, and root biomass) ± SE (*n* = 4) of *Salix* genotypes ‘Loden’ (gray) and ‘Tora’ (white) after 12 weeks of growth in two fertilizer (high [F+] and low [F-]) and two culture treatments (mixture [mix] and monoculture [mono]). **(B)** Comparisons of the plant biomass fractions (leaves, shoots, and roots, expressed as % dry weight) between ‘Loden’ and ‘Tora’ grown under the same treatments; with F+ and F-, and in mixture or monoculture.

The growth analysis results reported involved plant traits observed during the main growing season, i.e., referring to the period between the two destructive harvests, and with respect to whole-plant growth and biomass allocation. We found significant fertilizer treatment effects for total plant biomass and the proportional allocation of different plant parts (**Figure 1**; **Table 2**; Supplementary Table S1). Total biomass was significantly higher in the high fertilizer compared to low fertilizer treatment (*p* < 0.01) (**Figure 1A**; **Table 2**; Supplementary Table S2). In the low fertilizer treatment, ‘Tora’ showed a higher aboveground biomass in mixtures compared to monoculture, whereas ‘Loden’ showed the opposite pattern (Supplementary Table S2; *p* < 0.001). More biomass was allocated to roots under low fertilization (F-) compared to high fertilization (F+) (*p* < 0.001) (**Figure 1B**; Supplementary Table S1). The root biomass of ‘Loden’ plants was affected by the culture treatment (monoculture or mixture) only in the F+ treatment; this pattern was not apparent in the ‘Tora’ plants (G × C × F interaction effect, *p* < 0.05, Supplementary Table S1).

**Table 2:** Results from linear mixed effect model for growth parameters and N economy.

Factor	Leaf biomass (g)	Shoot biomass (g)	Root biomass (g)	AGB (g)	Total Biomass (g)	Leaf area (cm^2^)	RGR g (g^-1^ wk^-1^)	LAR (m^2^g^-1^)	LMR (g g^-1^)
F	^∗∗∗^	^∗∗∗^	^∗∗^	^∗∗∗^	^∗∗^	^∗∗∗^	^∗∗∗^	^∗∗^	ns
G	^∗^	^∗∗∗^	ns	^∗∗^	ns	ns	^∗∗∗^	^∗∗∗^	^∗^
C	ns	^∗^	ns	ns	ns	ns	ns	ns	ns
F × C	ns	ns	ns	ns	ns	ns	ns	ns	ns
F × G	ns	ns	ns	ns	ns	ns	ns	ns	ns
C × G	^∗^	ns	ns		ns	^∗^	ns	ns	ns
F × C × G	ns	ns	ns	ns	ns	ns	ns	ns	ns

**Factor**	**LAP (g m^-2^ wk^-1^)**	**SLA (m^2^ kg^-1^)**	**Root:shoot (g g^-1^)**	**SRL (m g^-1^)**	**SRA (cm^2^ g^-1^)**	**RMF (g g^-1^)**	**LN/LA (mmol N m^-2^)**	**LNP (g[mol N]^-1^ wk^-1^)**	**U_N_ (g g^-1^)**	**E_N,Y_ (g g^-1^)**

F	^∗∗∗^	ns	^∗∗^	ns	ns	^∗∗^	ns	^∗∗^	^∗∗∗^	^∗^
G	ns	ns	ns	ns	ns	ns	^∗∗^	^∗∗^	^∗^	^∗^
C	ns	ns	ns	ns	ns	ns	^∗^	ns	ns	ns
F × C	ns	ns	ns	ns	ns	ns	ns	ns	ns	ns
F × G	ns	ns	^∗^	ns	ns	ns	ns	ns	^∗∗^	ns
C × G	^∗^	ns	ns	ns	ns	ns	^∗∗^	^∗^	ns	ns
F × C × G	^∗^	ns	ns	ns	ns	^∗^	^∗^	^∗^	^∗∗^	^∗^

*Effects of fertilization (F), genotype (G) and mix-mono culture (C) on leaf, shoot, and root biomass (log_e_), aboveground biomass (AGB, log_e_), total biomass, leaf area, leaf area ratio (LAR, sqrt), leaf mass ration (LMR), leaf area productivity (LAP, sqrt), relative growth rate (RGR), specific leaf area (SLA), root to shoot ratio (Root:shoot, log_e_), specific root length (SRL), specific root area (SRA), root mass fraction (RMF), leaf N concentration (LN/LA), leaf N productivity (LNP), N uptake efficiency (U_*N*_) and yield specific N efficiency (E_*N,y*_) of *Salix* plants grown in a pot experiment in Sweden.*

*Symbols show result of a linear mixed effect model with significance levels: ns, not significant; ^∗^*p* < 0.05, ^∗∗^*p* < 0.01, ^∗∗∗^*p* < 0.001.*

**FIGURE 2:**
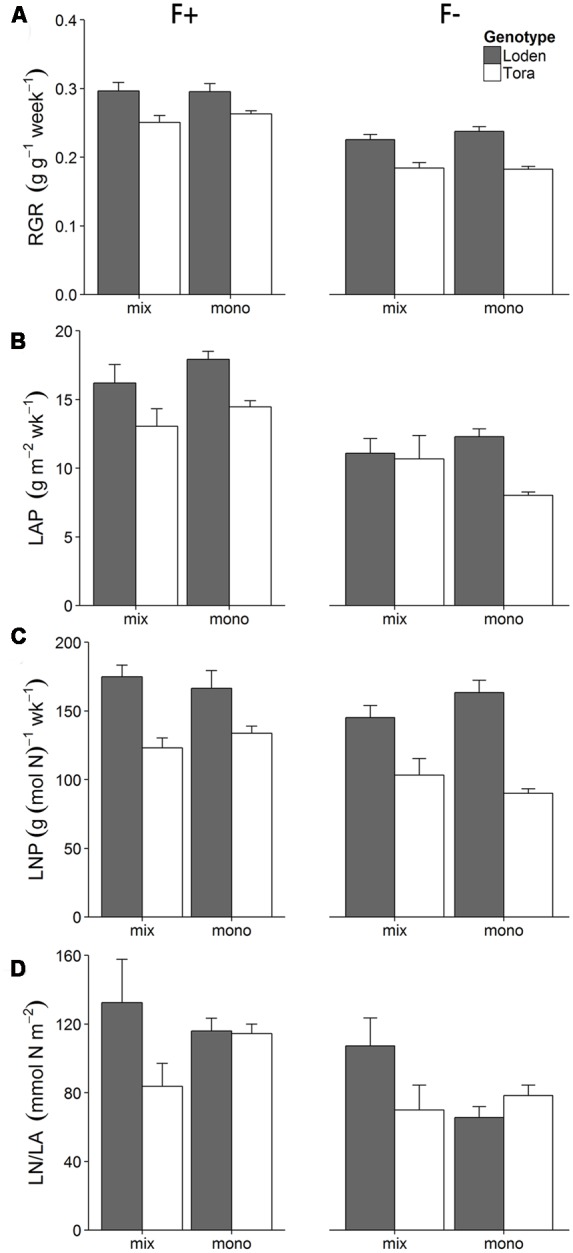
**Effect of two fertilization treatments (high [F+] and low [F-]) on (A)** relative growth rate (RGR), **(B)** leaf area productivity (LAP), **(C)** leaf N productivity (LNP) and **(D)** leaf N content (LN/LA) on two *Salix* genotypes ‘Loden’ (gray) and ‘Tora’ (white) grown in mixture (mix) and monoculture (mono). Values represent mean ± SE (*n* = 8).

The ‘Loden’ plants showed a higher relative growth rate (RGR) and leaf N productivity (LNP) than ‘Tora’ (**Figures 2A,C**). Higher fertilization dose increased RGR, LNP, LAP, and leaf area ratio (LAR) (**Figures 2A–C**, Supplementary Table S2). The culture treatment did not affect RGR and LAP (i.e., no significant main effect of C in **Table 2**), but triggered genotype-specific responses in LAP (i.e., the significant interaction effects in **Table 2**). For example, the LAP of ‘Loden’ was generally higher in monoculture compared to mixture, whilst ‘Tora’ showed a higher LAP in monoculture than mixture only in the high fertilizer treatment (**Figure 2B**, three-way interaction in **Table 2**, *p <* 0.05). The genotype-specific pattern in LAP was closely reflected by the corresponding pattern in LNP (e.g., definition of LAP in **Table 1**). Leaf N content (LN/LA) showed a different pattern compared to LNP (**Figure 2D**). For example, at high fertilization, LN/LA of ‘Loden’ was higher than ‘Tora’ only when grown in mixture. At low fertilization, LN/LA of ‘Loden’ was higher in the mixture than in the monoculture, but LN/LA of ‘Tora’ was similar in the two culture treatments (F × C × G, *p <* 0.05, **Table 2**; Supplementary Table S2).

### Nitrogen Economy

**FIGURE 3:**
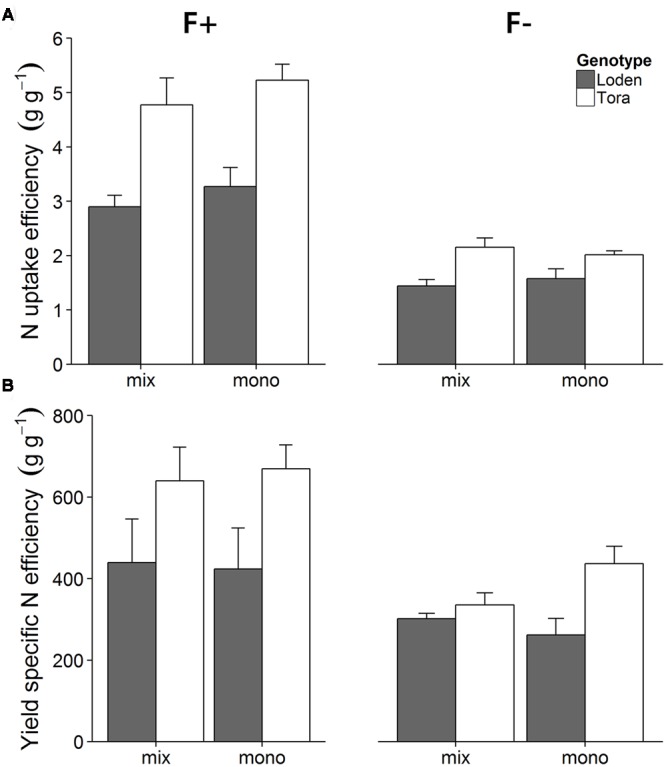
**Effects of two fertilizer treatments (F+, F-) on N uptake efficiency (A)** and yield specific N efficiency **(B)** of ‘Loden’ (gray) and ‘Tora’ (white) grown either in mixture (mix) or monoculture (mono). Values represent mean ± SE (*n* = 4).

In contrast to the growth analysis results, the N economy evaluation considers the whole life time of the plant with a focus on the potentially harvested product (here shoots that could be used for biomass) (Weih et al., 2011). Higher nutrient fertilization generally enhanced the N uptake efficiency (U_N_), and ‘Tora’ showed a higher N uptake efficiency than ‘Loden’ (**Figure 3A**; Supplementary Table S2, *p <* 0.001). In the high fertilization treatment, ‘Tora’ showed a higher N uptake efficiency in monoculture compared to the mixture, whereas the opposite pattern appeared in the low fertilizer treatment; in contrast to ‘Tora,’ ‘Loden’ showed a higher N uptake efficiency in monoculture compared to mixture in both fertilization treatments (F × C × G interaction effect, *p <* 0.01, Supplementary Table S2).

Yield specific N efficiency (E_N,y_, Supplementary Table S2) increased with fertilization, and ‘Tora’ showed a higher E_N,y_ than ‘Loden’ (**Figure 3B**, *p <* 0.05). The higher E_N,y_ in ‘Tora’ partly reflects the higher shoot biomass allocation of this genotype compared to ‘Loden’ (**Figure 1**). At high fertilization, yield specific N efficiency did not differ between mixture and monoculture for any of the genotypes. At low fertilization, ‘Tora’ had lower E_N,y_ when grown in mixture, whereas ‘Loden’ had higher E_N,y_ in the mixture. The differential responses to fertilization, genotype and mono-mix levels were reflected by a significant three-way interaction of the liner mixed effect model (**Table 2**).

### Aboveground Biomass, N Economy, and N Retention Efficiency at the Plant Community Level

**FIGURE 4:**
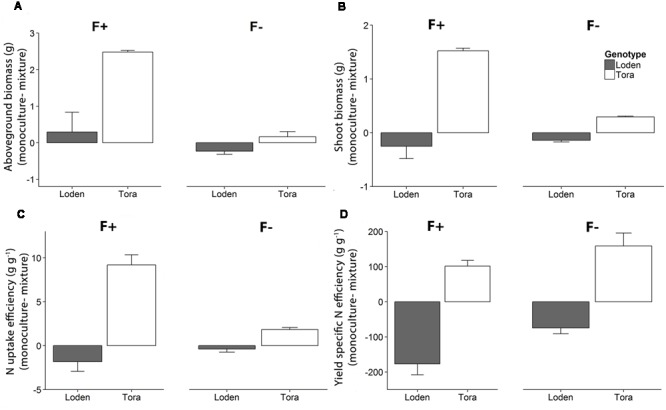
**Differences of community level (A)** aboveground biomass, **(B)** shoot biomass, **(C)** N uptake efficiency and **(D)** yield specific N efficiency between monocultures and mixtures, for ‘Loden’ (gray) and ‘Tora’ (white) grown under high (F+) and low (F-) fertilization levels. Negative values indicate higher biomass in the mixed community compared to the monoculture of ‘Loden’ or ‘Tora.’ Values represent mean values ± SE (*n* = 4–8).

At high fertilization, the community level (i.e., whole pot) aboveground biomass of ‘Tora’ and ‘Loden’ grown in monocultures was higher than the biomass attained by the mixtures (ANOVA, *p <* 0.05 for ‘Tora’ only, **Figure 4A**). For shoot biomass and N uptake efficiency (**Figures 4B,C**), a similar pattern was observed, although shoot biomass of ‘Loden’ tended to be higher in mixture compared to monoculture at high fertilization. Yield specific N efficiency (**Figure 4D**) of ‘Loden’ tended to be higher in mixture compared to the monoculture, whereas ‘Tora’ showed the opposite trend (statistically not significant). The community level N retention efficiency η (Supplementary Table S3) was significantly higher in the low fertilizer treatment (η = 0.78 ± 0.1 to 0.85 ± 0.03) than in the high fertilizer treatment (η = 0.46 ± 0.06 to 0.58 ± 0.06). In high fertilizer treatment, η of ‘Tora’ grown in monoculture tended to be higher (η = 0.58 ± 0.06) compared to the corresponding value when both genotypes grew in mixture (η = 0.46 ± 0.06).

### Diversity Effects

**FIGURE 5:**
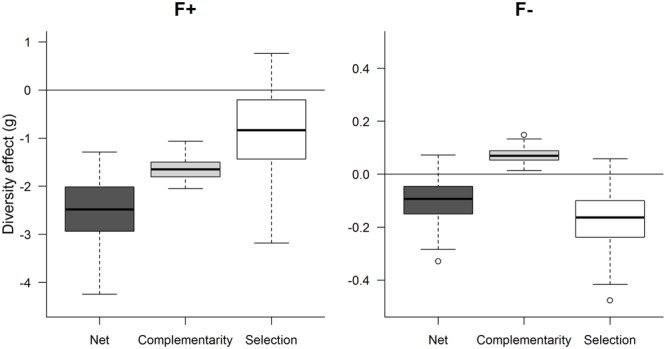
**Net, complementarity and selection effect for genotype mixture of ‘Loden’ and ‘Tora’ grown under high fertilization (F+) and low fertilization (F-).** The net effect was calculated as the sum of complementarity and selection effect. All means of 300 iterations of 7–8 replicated combinations are plotted with the help of a boxplot. The boxplot shows the median (50% quartile), the upper quartile (75%), and lower quartile (25%) as well as the upper and lower whisker representing the highest and lowest non-outlier data.

Net diversity, complementarity and selection effects were calculated based on the aboveground biomass per pot (**Figure 5**). Here, the averages across replicates of the 300 randomly selected combinations of monocultures and mixtures, for each treatment, were used to compare and statistically evaluate the diversity effects. At high fertilization, all net diversity effects were significantly negative and were driven by a strongly negative complementarity effect associated with a weakly negative selection effect. In contrast, at low fertilization, the significantly negative net diversity effect was driven by a significantly negative selection effect, whereas the complementarity effect was positive.

## Discussion

Our goals were to investigate the effect of mixture compared to monoculture on the productivity and N economy of young willow plants as a model in a controlled growth system, and to identify potential drivers of niche differentiation that can be transferred to other model systems. By using eco-physiological plant traits such as RGR, LAP, and N uptake efficiency, we aimed to frame our results within a broader field of studies dealing with plant community dynamics and the underlying mechanisms. Various studies investigating competitive growth performance in nutrient-poor and nutrient-rich environments have been conducted in grasslands (Aerts et al., 1990; Li and Watkinson, 2000; Nyfeler et al., 2009) and salt marshes (Morzaria-Luna and Zedler, 2014), but very few involve tree species (Aerts et al., 1991), especially none in *Salix* plants or between different *Salix* genotypes. While previous studies focused on the growth performance of *Salix* genotype mixtures in a given resource environment (McCracken et al., 2001, 2011; Begley et al., 2009), to our knowledge no study so far has tested the role of nutrient availability on growth performance and N economy in *Salix* genotype mixtures compared to monocultures.

### Higher Aboveground and Belowground Biomass in Mixture When Grown under Low Fertilization

We hypothesized that the biomass production of a two-component *Salix* genotype community should be greater compared to monoculture when nutrients are low. We found no clear evidence in support for this hypothesis, as a trend of higher biomass in the mixed community compared to one of its components (‘Loden’) grown in monoculture was not confirmed statistically. However, our results showed a higher aboveground biomass allocation as well as productivity in ‘Tora’ grown at low fertilization in mixture compared to monoculture, whereas the other genotype (‘Loden’) showed a lower aboveground biomass under low nutrient condition when grown in mixture compared to monoculture (**Figures 1** and **2**). This pattern is in line with the positive complementarity effect we found at low fertilization (**Figure 5**), indicating that niche differentiation occurred, which in the longer term (multiple years) could result in a changed population structure and a sampling effect (Tilman et al., 1997). ‘Loden’ is a genotype characterized by high LAR and differs from ‘Tora’ in terms of growth (lower shoot biomass, higher RGR) and plant N productivity (higher N productivity) (Weih and Nordh, 2002). In the temporal perspective of this study (i.e., one growing season from cutting to final harvest), it seems that ‘Loden’ is outcompeting ‘Tora’: ‘Loden’ increased its performance in mixture considerably more than ‘Tora’ in terms of the majority of traits assessed here (negative quantities in **Figure 4**), making ‘Loden’ appear as the superior competitor in this community. However, many of the traits assessed during the main growth period of this experiment indicated that ‘Tora’ could be more favored in mixtures at low fertilizer supply and in the longer term: The U_N_, LNP, and LAP of plants grown at low fertilizer level were similar or decreased in the mixture treatment in ‘Loden,’ but increased in ‘Tora,’ which caused a similar pattern also in RGR (**Figure 2**). Our results are partly supported by the results from a field study by Dillen et al. (2016), in which three *Salix* genotypes were grown in mixture and monoculture in the field for 4 years; differences in productivity between the genotypes grown in monoculture vs. mixture emerged here after 2 to 4 years of growth. We therefore speculate that in the longer term of more than 1 year, ‘Tora’ might perform better than ‘Loden’ in a two-component mixture grown at low nutrient supply. This expectation needs to be verified by long-term experimentation in the field. Although we cannot make robust conclusions regarding the relative performance of the investigated mixture in the long-term, we demonstrated here that different functional traits between the two genotypes resulted in different growth performance in mixture compared to monoculture.

Even though our results did not indicate different root biomass allocation between monoculture and mixture or between genotypes under low fertilization, both ‘Loden’ and ‘Tora’ showed a significantly higher root allocation under low resource availability, as expected due to root foraging for nutrients (Aerts et al., 1991; Canham et al., 1996). In a resource-limited greenhouse study, four seedlings of four tree species increased biomass allocation belowground under nutrient limitation (Canham et al., 1996), which is also in agreement with our results. In a competition experiment between two evergreen shrubs and the perennial grass *Molinia caerulea*, a strong competitive ability of the perennial grass, especially under high fertilization, was found (Aerts et al., 1991). The belowground biomass of *Molinia* increased when grown in mixture with both evergreen shrubs, but there was no belowground competition among the three species in the unfertilized treatment (Aerts et al., 1991). In our study, a different root allocation pattern between both genotypes grown in monoculture and mixture at high fertilization was found. On the one hand, ‘Loden’ invested more in roots when grown in a mixture with ‘Tora’ compared to the root investment in the corresponding monoculture. On the other hand, root allocation of ‘Tora’ was similar in mixture and monoculture. Root allocation was not significantly different between ‘Loden’ and ‘Tora’ when grown together under low fertilization, suggesting that there was little competition between the two genotypes in the current set up (as in Aerts et al., 1991). This may be the result of an oversized growth container or a too short growing period.

However, a decreased root allocation of ‘Tora’ in the mixture compared to monoculture under high fertilization (Supplementary Table S2; **Figure 1**) possibly indicates that the higher root growth of ‘Loden’ in the mixture might be due to pre-empting of resources to limit the root growth of ‘Tora.’

### *Salix* Genotypes Respond Differently in Aboveground Biomass at Plant Community Level

From ecological and agronomic perspectives it is most relevant to compare productivity of plants at the community level when grown in monoculture and mixture. Thus, we analyzed biomass data by aggregating the aboveground biomass data of the community components at pot scale. At the plant community level, the productivity of ‘Tora’ grown in monoculture was higher than the productivity in the mixed community at high fertilization (**Figure 4A**). However, ‘Loden’ showed no difference in aboveground biomass in monoculture compared to the plant community mixture. Thus, the aboveground biomass comparison and the negative net diversity as well as complementarity effect (**Figure 5**) indicate that ‘Loden’ is a better competitor than ‘Tora’ at high fertilization. This effect was less pronounced at low fertilization, where monocultures and mixtures had similar biomass and weakly negative diversity effects (despite positive complementarity, **Figure 5**). This result contradicts both our expectation (e.g., first hypothesis) and a study where grass and legume species grown in a grassland showed a higher productivity in mixtures compared to monoculture under low fertilization than under high fertilization (Nyfeler et al., 2009). In a greenhouse experiment, two salt marsh halophytes (*Triglochin concinna* and *Salicornia virginica*) showed contrasting competitive responses under both water and nitrogen availability – specifically, *Salicornia* responded negatively to *Triglochin* only at high fertilization (Morzaria-Luna and Zedler, 2014). Similar to *Salicornia*, in our study ‘Tora’ responded negatively to ‘Loden’ only at high fertilization, indicating contrasting competitive responses of the two *Salix* genotypes in our study.

The *Salix* genotypes used here are also employed in short rotation coppice systems for producing biomass for bioenergy. For this reason it is important to analyze patterns in the harvestable parts, i.e., shoot biomass, in monoculture and mixture. We found that ‘Tora’ had a significantly higher shoot biomass in monoculture than in mixture at high fertilization, whereas ‘Loden’ showed the opposite trend (**Figure 4**). It has been demonstrated that in fertile soil, broad-leaved grass show a competitive advantage by overgrowing narrow leaved grasses (Fynn et al., 2005). As ‘Loden’ has broader leaves compared to ‘Tora’ (Fransson et al., 2013), we expect ‘Loden’ to over-shade ‘Tora’ in mixtures, perhaps explaining why ‘Tora’ grows better when planted in monoculture (**Figure 4A**).

### N Economy of ‘Loden’ and ‘Tora’ Differs in Monoculture and Mixture at High and Low Fertilization

In our experiment and in line with Weih et al. (2014), both ‘Loden’ and ‘Tora’ showed a significantly higher pot-scale N retention efficiency (Supplementary Table S3), but lower N uptake efficiency at low fertilization, because low N availability decreased plant growth and total plant N (**Figure 3**; Supplementary Table S2). In addition, ‘Tora’ showed a stronger decrease in N uptake efficiency with decreasing fertilization than the genotype ‘Loden’; and the fertilization response of the two genotypes was significantly influenced also by the mixture vs. monoculture treatment. Significant three-way interaction in N economy traits thus indicates individual responses of the two genotypes to fertilization and culture environment (i.e., monoculture vs. mixture). For example at high fertilization, the mean plant N content of ‘Loden’ plants varied much more between monoculture and mixture compared to ‘Tora’ plants, whereas the total biomass of ‘Loden’ was similar when grown in monoculture and mixture. This indicates that the presence of ‘Tora’ plants probably influenced the N uptake of ‘Loden,’ but not its total biomass growth. Niche differentiation may thus have occurred in terms of N economy, but not resulted in different biomass growth within the short term of this study.

‘Tora’ plants were able to take up more N in monoculture under high N availability, whereas under low N availability N uptake was higher in mixtures compared to the corresponding monoculture, which partly confirms our second hypothesis. Utilization of the acquired plant N is reflected by the N productivity and the yield specific N efficiency, the latter increased with fertilization as expected (Weih and Nordh, 2002). Interestingly, at low fertilization, ‘Loden’ had a similar yield specific N efficiency in monoculture and mixture, while ‘Tora’ plants showed a decrease in yield specific N efficiency when grown in mixture compared to monoculture. This decrease in yield specific N efficiency in the mixture might be due to an additional shading from the larger leaf area of the neighboring ‘Loden’ plants, which again indicates emerging niche differentiation of community components (here genotypes) in terms of N economy, accomplished by the differential expression of functional traits in the community components.

### Experimental Design and Future Developments

Interpreting plant competition experiments is often limited by factors such as the duration of the experiment and weather conditions (Aerts et al., 1991). The present experiment was designed using the two genotypes with different phenological characteristics and N economy (Weih and Nordh, 2002), grown under a transparent roof to control water and N inputs. Despite cool temperatures during the plant establishment (mean temperature 14.9 ± 5.1°C, relative humidity 67.9 ± 17.6%) resulted in late sprouting, plants grew well. In this study we also applied an irrigation treatment (see Materials and Methods), which however, did not induce any changes in biomass allocation (Supplementary Table S4), growth rate (e.g., RGR) and leaf traits (e.g., LAR, LMR, and LAP). Studies of niche differentiation with regard to functional traits important for water economy, thus require experimental treatments that expose plants to much stronger water stress than we had been able to perform in this study.

## Conclusion

Our results demonstrate that taxonomically closely related genotypes with different functional traits perform differently in mixture than in monoculture even in a short-term experiment, providing some evidence that niche differentiation has occurred among the community components (genotypes) investigated here. Considering functional traits, we found that only at low nutrient availability and in a mixed community, one genotype performed better than the other, thanks to its higher LAP, LNP, and N uptake efficiency. In contrast, monocultures performed consistently better at high nutrient availability. Thus, the specific combination of individual (genotype) functional traits achieved when genotypes were grown in mixture, resulted in niche differentiation and improved community growth as well as nitrogen use efficiency especially under low resource availability.

## Author Contributions

SH: Conception and design, collection and assembly of data, data analysis and interpretation, and manuscript writing; PF: Conception and design, data interpretation, and manuscript writing; IP-R: Collection and assembly of data, and data analysis; SM: Conception and design, data interpretation, and manuscript writing; MW: Conception and design, data interpretation, and manuscript writing. All authors reviewed the manuscript.

## Conflict of Interest Statement

The authors declare that the research was conducted in the absence of any commercial or financial relationships that could be construed as a potential conflict of interest.
