# Supplementary material for: Two Salix Genotypes Differ in Productivity and Nitrogen Economy When Grown in Monoculture and Mixture
Source: Front Plant Sci. 2017 Feb 21;8:231. doi: 10.3389/fpls.2017.00231 (PMC5318404; doi:10.3389/fpls.2017.00231)
Supplement: Supplementary file 1 [file Table_1.docx]

**Supplementary material**

Table S1. ANCOVA of the effect of fertilizer (F), genotype (G) and mix- mono culture (C) treatment on root biomass with total biomass as covariate.

| Factor | Root biomass |
| --- | --- |
| Total biomass | *** |
| G | * |
| C | ns |
| F | *** |
| Total biomass x G | *** |
| Total biomass x C | ns |
| G*C | ns |
| Total biomass x F | ns |
| G*F | ns |
| C*F | ns |
| Total biomass x G x C | *** |
| Total biomass x G x F | ns |
| Total biomass x C x F | ** |
| G x C x F | * |
| Total biomass x G x C x F | ns |

Symbols indicate level of significance: ns=not significant, * = p< 0.05, ** = p<0.01, *** = p< 0.001
