# Supplementary material for: Two Salix Genotypes Differ in Productivity and Nitrogen Economy When Grown in Monoculture and Mixture
Source: Front Plant Sci. 2017 Feb 21;8:231. doi: 10.3389/fpls.2017.00231 (PMC5318404; doi:10.3389/fpls.2017.00231)
Supplement: Supplementary file 2 [file Table_2.docx]

Table S2. Growth parameters, N uptake efficiency (U_N_) and yield specific N efficiency (E_N,y_) for two *Salix* genotypes (initial plant biomass in parentheses) grown in two different fertilizer (F+, F-) and culture (mono, mix) treatments in Sweden. Values represent means ± SE (n= 4-8).

| \| Genotype (intital  m_plant_, g) \| Treat-  ment \| Leaf  biomass  (g) \| Shoot biomass (g) \| Root biomass  (g) \| Root : shoot ratio  (g g^-1^) \| AGB  (g) \| Total Biomass  (g) \| LMR (g g^-1^) \| RMF (g g^-1^) \| Leaf area  (cm^2^) \| RGR (g g^-1^wk^-1^) \| \| --- \| --- \| --- \| --- \| --- \| --- \| --- \| --- \| --- \| --- \| --- \| --- \| \| Loden \| F+mono \| 0.92 ± 0.10 \| 0.59 ± 0.09 \| 0.83 ± 0.18 \| 1.71 ± 0.19 \| 1.51 ± 0.19 \| 2.22 ± 0.49 \| 0.65 ± 0.03 \| 0.37 ± 0.03 \| 114.1 ± 14.3 \| 0.30 ± 0.018 \| \| (0.05) \| F-mono \| 0.36 ± 0.03 \| 0.15 ± 0.02 \| 0.48 ± 0.05 \| 3.79 ± 1.11 \| 0.52 ± 0.05 \| 1.00 ± 0.10 \| 0.71 ± 0.03 \| 0.47 ± 0.05 \| 44.4 ± 4.3 \| 0.24 ± 0.004 \| \|  \| F+mix \| 0.66 ± 0.07 \| 0.38 ± 0.08 \| 0.83 ± 0.20 \| 1.81 ± 0.15 \| 1.08 ± 0.11 \| 2.03 ± 0.29 \| 0.63 ± 0.07 \| 0.38 ± 0.04 \| 81.0 ± 8.8 \| 0.30 ± 0.007 \| \|  \| F-mix \| 0.31 ± 0.03 \| 0.13 ± 0.01 \| 0.38 ± 0.03 \| 2.90 ± 0.35 \| 0.45 ± 0.04 \| 0.86 ± 0.09 \| 0.67 ± 0.02 \| 0.46 ± 0.04 \| 39.1 ± 3.9 \| 0.23 ± 0.001 \| \| Tora \| F+mono \| 0.91 ± 0.04 \| 0.85 ± 0.05 \| 0.87 ± 0.07 \| 1.09 ± 0.11 \| 1.76 ± 0.09 \| 2.64 ± 0.10 \| 0.53 ± 0.02 \| 0.33 ± 0.02 \| 112.1 ± 5.6 \| 0.26 ± 0.003 \| \| (0.09) \| F-mono \| 0.31 ± 0.01 \| 0.21 ± 0.01 \| 0.48 ± 0.01 \| 2.39 ± 0.13 \| 0.52 ± 0.01 \| 1 .00± 0.01 \| 0.59 ± 0.02 \| 0.47 ± 0.01 \| 38.5 ± 1.5 \| 0.18 ± 0.001 \| \|  \| F+mix \| 0.83 ± 0.10 \| 0.76 ± 0.10 \| 0.76 ± 0.05 \| 1.13 ± 0.11 \| 1.59 ± 0.19 \| 2.33 ± 0.23 \| 0.53 ± 0.04 \| 0.34 ± 0.02 \| 109.0 ± 12.6 \| 0.25 ± 0.009 \| \|  \| F-mix \| 0.40± 0.04 \| 0.21 ± 0.02 \| 0.47 ± 0.04 \| 3.01 ± 0.38 \| 0.60 ± 0.05 \| 1.04 ± 0.11 \| 0.66 ± 0.03 \| 0.47 ± 0.02 \| 41.3 ± 3.8 \| 0.18 ± 0.011 \| \|  \|  \|  \|  \|  \|  \|  \|  \|  \|  \|  \|  \| \| Genotype \| Treat-  ment \| LAR (m^2^ kg^-1^) \| LAP (g m^-2^ wk^-1^) \| SLA (m^2^ kg^-1^) \| SRL  (m g^-1^) \| SRA (cm^2^ g^-1^) \| Total  Plant N (mg) \| LN/LA (mmol N m^-2^) \| LNP (g [mol N] ^-1^ wk^-1^) \| E_N,y_ (g g^-1^) \| U_N_ (g g^-1^) \| \| Loden \| F+mono \| 7.83 ± 0.15 \| 17.9 ± 0.5 \| 13.03 ± 0.98 \| 80.6 ± 14.2 \| 186.9 ± 11.7 \| 23.75 ± 2.92 \| 116.0± 6.4 \| 166.5 ± 11.2 \| 423.4 ± 87.3 \| 3.27 ± 0.30 \| \|  \| F-mono \| 8.85 ± 0.32 \| 12.3 ± 0.5 \| 12.58 ± 0.47 \| 81.8 ± 7.3 \| 224.7 ± 23.0 \| 5.76 ± 0.93 \| 65.6 ± 5.5 \| 163.5 ± 7.8 \| 261.6 ± 35.5 \| 1.58 ± 0.15 \| \|  \| F+mix \| 8.08 ± 0.35 \| 16.2 ± 1.2 \| 12.84 ± 1.30 \| 90.8 ± 22.4 \| 234.8 ± 48.4 \| 14.77 ± 2.53 \| 132.6 ± 21.8 \| 175.0 ± 7.3 \| 439.5 ± 92.3 \| 2.90± 0.18 \| \|  \| F-mix \| 9.22 ± 0.64 \| 11.1 ± 0.9 \| 13.13 ± 1.18 \| 110.8 ± 10.6 \| 225.1 ± 11.9 \| 5.04 ± 0.67 \| 107.4 ± 14.0 \| 145.2 ± 7.5 \| 301.7 ± 11.2 \| 1.44 ± 0.10 \| \| Tora \| F+mono \| 6.44 ± 0.12 \| 14.5 ± 0.4 \| 12.51 ± 0.37 \| 111.0 ± 13.3 \| 262.8 ± 29.6 \| 23.06 ± 2.52 \| 114.5 ± 4.8 \| 133.7 ± 4.5 \| 669.3 ± 51.2 \| 5.23 ± 0.25 \| \|  \| F-mono \| 7.46 ± 0.23 \| 8.0 ± 0.2 \| 12.53 ± 0.36 \| 104.6 ± 7.7 \| 248.8 ± 15.2 \| 5.86 ± 0.61 \| 78.4 ± 5.2 \| 90.1 ± 2.8 \| 436.4 ± 37.2 \| 2.02 ± 0.06 \| \|  \| F+mix \| 7.12 ± 0.40 \| 13.1 ± 1.1 \| 15.00 ± 2.03 \| 86.1 ± 12.8 \| 222.9 ± 21.5 \| 21.61 ± 2.84 \| 83.7 ± 11.7 \| 123.1 ± 6.2 \| 639.6 ± 71.7 \| 4.77 ± 0.43 \| \|  \| F-mix \| 7.44 ± 0.43 \| 10.7 ± 1.5 \| 12.32 ± 0.97 \| 100.4 ± 16.8 \| 242.2 ± 17.9 \| 7.43 ± 0.71 \| 70.0 ± 12.5 \| 103.2 ± 10.5 \| 335.4 ± 25.9 \| 2.16 ± 0.15 \| |
| --- | --- | --- | --- | --- | --- | --- | --- | --- | --- | --- | --- | --- | --- | --- | --- | --- | --- | --- | --- | --- | --- | --- | --- | --- | --- | --- | --- | --- | --- | --- | --- | --- | --- | --- | --- | --- | --- | --- | --- | --- | --- | --- | --- | --- | --- | --- | --- | --- | --- | --- | --- | --- | --- | --- | --- | --- | --- | --- | --- | --- | --- | --- | --- | --- | --- | --- | --- | --- | --- | --- | --- | --- | --- | --- | --- | --- | --- | --- | --- | --- | --- | --- | --- | --- | --- | --- | --- | --- | --- | --- | --- | --- | --- | --- | --- | --- | --- | --- | --- | --- | --- | --- | --- | --- | --- | --- | --- | --- | --- | --- | --- | --- | --- | --- | --- | --- | --- | --- | --- | --- | --- | --- | --- | --- | --- | --- | --- | --- | --- | --- | --- | --- | --- | --- | --- | --- | --- | --- | --- | --- | --- | --- | --- | --- | --- | --- | --- | --- | --- | --- | --- | --- | --- | --- | --- | --- | --- | --- | --- | --- | --- | --- | --- | --- | --- | --- | --- | --- | --- | --- | --- | --- | --- | --- | --- | --- | --- | --- | --- | --- | --- | --- | --- | --- | --- | --- | --- | --- | --- | --- | --- | --- | --- | --- | --- | --- | --- | --- | --- | --- | --- | --- | --- | --- | --- | --- | --- | --- | --- | --- | --- | --- | --- | --- | --- | --- | --- | --- | --- | --- | --- | --- | --- | --- | --- | --- | --- | --- |
