# Supplementary material for: Two Salix Genotypes Differ in Productivity and Nitrogen Economy When Grown in Monoculture and Mixture
Source: Front Plant Sci. 2017 Feb 21;8:231. doi: 10.3389/fpls.2017.00231 (PMC5318404; doi:10.3389/fpls.2017.00231)
Supplement: Supplementary file 3 [file Table_3.docx]

Table S3. N retention efficiency (ƞ) at community (pot) level for two *Salix* genotypes grown in two different fertilizer (F+, F-) and culture (monoculture, mixture) treatments. Values represent means ± SE (n= 8).

| Genotype | Fertilization  treatment | Culture  treatment | ƞ |
| --- | --- | --- | --- |
| Loden | F+ | mono | 0.48 ± 0.06 |
|  | F- | mono | 0.78 ± 0.11 |
| Tora | F+ | mono | 0.58 ± 0.06 |
|  | F- | mono | 0.85 ± 0.03 |
| Loden and | F+ | mix | 0.46 ± 0.04 |
| Tora | F- | mix | 0.80 ± 0.10 |
