# Supplementary material for: Two Salix Genotypes Differ in Productivity and Nitrogen Economy When Grown in Monoculture and Mixture
Source: Front Plant Sci. 2017 Feb 21;8:231. doi: 10.3389/fpls.2017.00231 (PMC5318404; doi:10.3389/fpls.2017.00231)
Supplement: Supplementary file 4 [file Table_4.docx]

Table S4. Results from linear mixed effect model for growth parameters and N economy. Effects of water (W), fertilization (F), genotype (GT) and mix- mono culture (C) on various growth parameters and N economy.

| **Factor** | **Leaf biomass** | **Shoot biomass** | **Root biomass** | **AGB** | **Total biomass** | **Leaf area** | **LAR** |
| --- | --- | --- | --- | --- | --- | --- | --- |
| W | ns | ns | ns | ns | ns | ns | ns |
| F | *** | * | ns | ** | ns | ** | ns |
| GT | ns | ns | ns | ns | ns | ns | *** |
| C | ns | ns | ns | ns | ns | ns | ns |
| W x F | ns | ns | ns | ns | ns | ns | ns |
| W x GT | ns | ns | ns | ns | ns | ns | ** |
| F x GT | ns | * | ns | ns | ns | ns | ** |
| W x C | ns | ns | ns | ns | ns | ns | ns |
| F x C | ns | ns | ns | ns | ns | ns | ns |
| GT x C | ns | ns | ns | ns | ns | ns | ns |
| W x F x GT | ns | ns | ns | ns | ns | ns | * |
| W x F x C | ns | ns | ns | ns | ns | ns | ns |
| W x GT x C | ns | ns | ns | ns | ns | ns | ns |
| F x C x GT | ns | ns | ns | ns | ns | ns | ns |
| W x F x C x GT | ns | ns | ns | ns | ns | ns | ns |

| Factor | **LMR** | **LAP** | **RGR** | **SLA** | **Root:shoot** | **SRL** | **SRA** |
| --- | --- | --- | --- | --- | --- | --- | --- |
| W | ns | ns | ns | ** | ns | ns | ns |
| F | ns | ******* | ******* | * | ns | ns | ns |
| GT | ns | ns | ***** | ** | ns | ns | ns |
| C | ns | ns | ns | ns | ns | ns | ns |
| W x F | ns | * | ns | ** | ns | ns | ns |
| W x GT | ns | * | ns | ** | ns | ns | ns |
| F x GT | ** | ** | * | *** | ns | ns | ns |
| W x C | ns | ns | ns | * | ns | ns | ns |
| F x C | ns | ns | ns | ns | ns | ns | ns |
| GT x C | ns | ****** | ***** | ** | * | ns | ns |
| W x F x GT | ** | ** | * | ** | ns | ns | ns |
| W x F x C | ns | ns | ns | ns | ns | ns | ns |
| W x GT x C | ns | ns | ns | ** | ns | ns | ns |
| F x C x GT | ns | ****** | * | ** | ns | ns | ns |
| W x F x C x GT | ns | * | ns | * | ns | ns | ns |
| Transformations of variables |  | Sqrt |  | log |  |  |  |

| Factor |  | **RMF** | **LN/LA** | **LNP** | **U_N_** | **E_N,y_** |
| --- | --- | --- | --- | --- | --- | --- |
| W |  | ns | ns | ns | ns | ns |
| F |  | ns | ns | ns | * | ns |
| GT |  | ns | ns | ns | ns | ns |
| C |  | ns | ns | ns | ns | ns |
| W x F |  | ns | ns | ns | ns | ns |
| W x GT |  | ns | ns | ns | ns | ns |
| F x GT |  | ns | ns | ns | ns | ns |
| W x C | | ns | ns | ns | ns | ns |
| F x C |  | ns | ns | ns | ns | ns |
| GT x C | | ns | ns | ns | ns | ns |
| W x F x GT | | ns | ns | ns | ns | ns |
| W x F x C | | ns | ns | ns | ns | ns |
| W x GT x C | | ns | ns | ns | ns | ns |
| F x C x GT | | ns | ns | ns | ns | ns |
| W x F x C x GT | | ns | ns | ns | ns | ns |

Symbols indicate level of significance: ns=not significant, * = p< 0.05, ** = p<0.01, *** = p< 0.001
